# Gross Anatomy—A Brief Resume of the Anatomy of the Kidneys

**Published:** 1901-08

**Authors:** Byron H. Daggett

**Affiliations:** Buffalo, N. Y.


### Gross Anatomy—A Brief Resume of the Anatomy of the Kidneys.

A LECTURE DELIVERED TO THE CLASS OF NURSES AT BUFFALO HOSPITAL OF THE SISTERS OF CHARITY, APRIL, 1901.

By BYRON H. DAGGETT, M. D., Buffalo, N. Y.

THE kidneys are symmetrical organs, located in the lumbar region, behind the peritoneum, invested by a fibrous coat, and surrounded by some adipose tissue.

They extend from about the 11th or 12th rib downward toward the crest of the ileum. They are bean-shaped, with a central concavity, the deep portion of which is called the hilum. They weigh from 4 to 6 ounces.

The fibrous coat extends into the hilum, lines the pelvis and covers the calices and blood-vessels, which enter the kidney at this point. The cavity at the hilum is formed by the dilated origin or funnel of the ureter and is called the pelvis. The kidney is composed of two distinctly marked portions, called the cortical and medullary divisions. The cortical substance is reddish and grandular, and one-sixteenth of an inch in thickness. It is the outer portion of the organ. The surface of the kidney is lobulated by the arrangement of the superficial vessels. The medullary, or inner portion is firmer and darker red in color than the cortical and is made up of tubules, and is sometimes called the tubular portion. The kidney is made up of investing membrane, tubules, bloodvessels, lymphatics, nerves and connective tissue; a complex arrangement of structure, whose function is to separate the urine from the blood.

The physiological and minute anatomy of the kidney is exceedingly complex and I will not attempt its description.

The function of the kidneys consists in the secretion of the urine. There is no satisfactory explanation of the secretion and excretion of urine, in all its details. Flint says that in the present state of our knowledge we must regard the excrementitious constituents of the urine as formed essentially in the system at large, and merely separated from the blood by the kidneys.

*Physical characteristics of the urine.*—The amount excreted by a healthy person, who eats and drinks moderately, varies between 1400 and 1600 c.c. or an average of 1500 c.c. in 24 hours. The amount varies with the time of day, with the activity of the individual, with the amount of fluid imbibed, and with the amount of perspiration, etc.

*Color.*—The peculiar color of urine is due to a nitrogenised principle known by a variety of names, viz.: urrosacine, urochrome, urohematine, uroxanthine, and purpurine. Its ultimate composition is not settled, although it contains C.O.H.N. and probably iron. It is supposed to be much like the coloring matter of the blood, and probably formed from hematine, the coloring matter of that fluid.

Methylene blue passes readily through the kidneys, administered in the mouth, giving a decided indigo-blue color. Bile, blood, and the like, color the urine characteristically, but these questions belong to another branch of medicine.

*Odor.*—The odor of fresh urine is slightly aromatic; the cause of this odor is unknown. The temperature of fresh urine as it passes is nearly 100° F. As it cools it loses its aromatic odor and becomes urinous and then ammoniacal. The use of certain foods and medicines change remarkably the odor. For example, asparagus, cauliflower, turpentine, cubebs and saffron.

*Transparency.*—Normal or physiological urine is always clear and transparent, and if it is not transparent its consideration belongs to the diagnostician or pathologist—our study is physiology.

*Reaction.*—Normal urine is acid in reaction, and its acidity is due especially to the acid alkali-phosphates. It may be due to the presence of a free organic acid, but the rôle played by free acid is unimportant, and the fact of its presence is a mooted question. Sometimes directly after a meal the urine is alkaline, but this phenomenon soon disappears, and it has no clinical significance.

The alkaline carbonates and salts of the vegetable acids taken into the stomach render the urine alkaline. It may seem curious that both alkalies and vegetable acids have this effect, but physiological chemistry accounts for this phenomenon.

The reaction of urine is usually tested by blue and red litmus paper. You must note whether the urine is alkaline when voided, and after standing for a time. Note also whether the alkalinity, if present, be due to the volatile, (ammo. carb. from breaking up of urea), or the fixed alkalies (alkali-phosphates) taken into the body as food or medicine. To determine this point, the red litmus, turned blue by the urine if exposed for a time to warm air, will return to its red color, but if the reaction is from fixed alkalies it will remain blue after drying. Sometimes the same urine will turn blue litmus red, and red litmus blue. This phenomenon is called amphoteric, and has no satisfactory explanation and no symptomatic value.

Bradshaw, of Liverpool, maintains that litmus could not be employed as an indicator in the estimation of acidity of the urine, because solutions of the salts of phosphoric acid are never neutral to litmus; that if they were not distinctly acid or alkaline they were amphoteric.

Bradshaw advocated the use of phenol-phthaline as an indicator of acidity, and maintained that objections to its use are not valid. The teaching is that acidity of the urine is diminished after meals. Bradshaw found, after making 160 estimates of the acidity of urine, that in health the acidity followed a fairly regular curve; high during sleep, it fell rapidly after "getting up" and remained at a minimum until about noon, after which it rose steadily and reached a maximum about 9 p.m.

He claims that the amount of acid excreted is apparently diminished or increased by a more or less bulk of water present, and that alkalies and other diuretics only apparently lessen the amount of acid by dilution and by combination.

*Solid Ingredients.*—The amount of solids excreted in twenty-four hours is generally 60 to 70 grams. If we find a small amount of solids, say 20 grams, and the quantity of water increased, it indicates hydruria. If we find a large amount of water and a normal or lesser amount of solids it is called polyuria.

Urinary insufficiency is a condition in which the amount of solids is abnormally diminished, without reference to the bulk of water. If there is morbidly diminished secretion it is called oliguria.

To estimate the amount of solids passed in twenty-four hours, multiply the decimal of the specific gravity by the coefficient 2.33 which gives the amount of solids in 1000 c.c. If the urine—twenty-four hours' product—is measured by ounces multiply the decimal of the specific gravity by the number of ounces and this sum by 1.1; this gives you the total solids in grains.

Solids of human urine, according to Flint: (1) urea; (2) urate of soda, neutral and acid; urate of ammonia, neutral and acid, small quantity; urate of potassa, trace; urate of lime, trace; urate of magnesia, trace; hipperate of soda, potassa and lime; lactate of soda, potassa and lime; creatine; creatinine; oxalate of lime; xanthine—indican, uroindican; margarine, olein and other fatty matters; chloride of sodium; chloride of potassium; hydrochlorate of ammonia; sulphate of soda, potassa and lime; phosphate of soda, neutral and acid; phosphate of lime, acid and basic; phosphate of magnesia; ammonium—magnesium phosphate; silicic acid; urrosacin, coloring matter; mucus; gases.

These are the normal constituents of the urine: total solids, 4.3 to 4.6 per cent.; urea, 2.5 to 3.2 per cent.; uric acid, 0.03 to 0.05 per cent.; creatinine, 0.036 to 0.062 per cent.; hippuric acid, 0.02 to 0.06 per cent.; chlorides, 0.7 to 0.8 per cent.; earthy phosphates, 0.07 to 0.08 per cent.; phosphoric acid, 0.19 to 0.22 per cent.; sulphuric acid, 0.10 to 0.17 per cent.

These tables show that urea and the chlorides are in a large percentage, so that the specific gravity is mostly due to these substances.

The other substances are present in small quantities and some only in traces and their presence or absence changes the gravity to a slight degree. The gases have slight importance.

Urea is the most constant of the urinary constituents, and exists in the greatest quantity. 30 to 40 grams are secreted in twenty-four hours. Upon a flesh diet more is excreted than upon vegetable diet. It is a nitrogenous substance, and is derived from the waste of nitrogenised principles.

Urea is found in several of the fluids and solids—muscles and liver—of the body. It is one of the few organic principles that can be produced synthetically by the chemist.

Uric acid is a constant constituent in the urine of the carnivora. It is sparingly soluble in water and totally so in alcohol and ether, so that it exists in small quantities as a free acid, but is generally found in combination as urates. It is a dibasic acid, forms both neutral and acid salts. It bears a relation, quantitative relation, with urea—1 to 30.

The remaining normal organic constituents of the urine have little value in general diagnosis, for the reason it is so difficult to identify and separate them, and the rôle they play in the human economy is an incomplete chapter.

*Normal Inorganic Constituents.*—They are taken into the system with the food.

*Chlorides.*—Next to urea the chlorides are the principal constituents of the urine. The chlorides are almost entirely the sodic and potassic salts. They diminish in repose of the body, in all acute febrile diseases, and in chronic affections. They increase with a salty diet, with energetic bodily or mental exercise, in intermittent fever and with dropsy.

*Phosphates.*—3.5 grams of phosphoric acid in combination are excreted daily. The phosphates increase with the introduction of phosphatic compounds; in acute febrile conditions and with the use of abundant animal diet. They decrease in urine of low specific gravity; in kidney and heart diseases and in disorders of digestion and chronic diseases of the brain.

*Sulphates.*—Are the neutral sulphates (salts) of potassium and sodium. They increase by an increased exhibition of the sulphur compounds and from an exclusive flesh diet, and in certain febrile processes with excessive excretion of urea. They decrease with an exclusive vegetable diet.

*Abnormal constituents of the urine.*—I will pass for the present the abnormal and accidental constituents, including sediments. They are albumin, sugar, leucine, tyrocine, certain coloring matters, bile acids and accidental substances. The kidneys excrete normal, abnormal, pathological, and accidental substances.

The practical lesson to be learned from this brief outline of the renal function may be stated as follows: You, as nurses, are to know the amount of urine passed by the patient in twenty-four hours, its color, its odor, its gravity, its reaction, and its transparency. You should supply yourselves with a urinometer, a small open-mouthed bottle, tightly corked, containing slips of both red and blue litmus. The amount, the gravity, the reaction and clearness of the clinical urine are therapeutic guide posts along the medical highway.

723 ELLICOTT SQUARE.

